# Training in a Hot Environment Fails to Elicit Changes in the Blood Oxidative Stress Response

**DOI:** 10.5114/jhk/161586

**Published:** 2023-04-20

**Authors:** Cassie M. Williamson-Reisdorph, Tiffany S. Quindry, Katherine S. Christison, Shae C. Gurney, Kathryn G. Tiemessen, John Cuddy, Walter Hailes, Dustin Slivka, Brent C. Ruby, John C. Quindry

**Affiliations:** 1 School of Integrative Physiology and Athletic Training, University of Montana, Missoula, MT, USA.

**Keywords:** heat physiology, hyperthermic exercise, free radicals, redox balance, recovery

## Abstract

Environmental temperature can impact exercise-induced blood oxidative stress; however, the effects of heat acclimation on this response have not been fully elucidated. The purpose of the study was to investigate the effects of hot (33°C) and room temperature (20°C) environments on post-exercise blood oxidative stress responses following 15 temperature acclimation sessions. Untrained participants (n = 38, 26 ± 7 years, VO_2peak_ = 38.0 ± 7.2 years) completed 15 temperature acclimation sessions of a cycling bout at an intensity perceived as “hard” in either a hot (33°C) or room temperature (20°C) environment. Pre and post acclimation exercise tolerance trials were conducted, which involved cycling at 50% W_peak_ for one hour. Blood sampling occurred before exercise, immediately after, two hours, and four hours after the exercise tolerance trials. Blood samples were analyzed for oxidative stress markers including lipid hydroperoxides, 8-isoprostanes, protein carbonyls, 3-nitrotyrosine, ferric-reducing ability of plasma, and Trolox-equivalent antioxidant capacity. Exercise-dependent increases were observed in lipid hydroperoxides, Trolox-equivalent antioxidant capacity, and ferric-reducing ability of plasma (p < 0.001). Considering exercise-induced elevations in markers of blood oxidative stress, there were no differences observed between environmental temperatures before or after the acclimation training period.

## Introduction

Oxidative stress during exercise occurs when there is excessive production of highly reactive molecules with unpaired electrons, termed reactive oxygen species (ROS). ROS initiate chain reactions resulting in damage to cellular components, including lipids, protein, and antioxidant fortifications (Powers et al., 2011; Souza-Silva et al., 2016). While chronic elevations in oxidative stress are associated with pathophysiological outcomes (e.g., cancer, heart diseases), acute perturbations due to ROS production are necessary to initiate many of the beneficial adaptive responses to exercise (Gomez-Cabrera et al., 2008; Powers et al., 2011). Indeed, following an acute bout of exercise, the generated ROS can upregulate cellular defenses such as endogenous antioxidant enzymes (Gomez-Cabrera et al., 2008; Quindry et al., 2013; Rudarli Nalcakan et al., 2021). Previous investigations provide preliminary evidence that ambient temperature impact the oxidative stress response independent of exercise and in response to acute hyperthermic exercise (Laitano et al., 2010; McAnulty et al., 2005; Quindry et al., 2013; Sureda et al., 2015). For instance, treadmill running at low intensity (50% VO_2max_) within hyperthermic conditions (35°C, 70% relative humidity) resulted in a significant elevation in plasma F_2_-isprostanes, indicating cellular lipid damage (McAnulty et al., 2005). Additionally, there is evidence to support that six minutes of one-legged knee extensor exercise leads to an elevation in antioxidant capacity (Laitano et al., 2010).

Although blood oxidative stress responses to exercise in hyperthermic conditions have been investigated, the effects of hyperthermic exercise training on the blood oxidative stress response remains understudied. To date, one investigation has examined the effects of hyperthermic exercise training consisting of four-week high-intensity interval training (HIIT) on the blood oxidative stress response to acute exercise in trained male subjects. The investigation determined that biomarkers of lipid peroxidation were elevated following the training period; however, markers of protein damage (protein carbonyls) were blunted by hyperthermic exercise training (Souza-Silva et al., 2016). Given the importance of ROS production on exercise adaptations, additional research is required to understand the impact of hyperthermic exercise training on the blood oxidative stress response to various modalities and/or intensities of exercise. Therefore, the primary purpose of the current investigation was to examine the effects of three-week heat acclimation training on the blood oxidative stress response to an acute bout of moderate-intensity aerobic exercise. A strategic panel of blood oxidative stress biomarkers was selected to quantify lipid damage (lipid hydroperoxides, LOOH; 8-isoprostane, 8-ISO), protein damage (protein carbonyls, PC; 3-nitrotyrosine, 3-NT), and antioxidant status (Trolox equivalent antioxidant capacity, TEAC; Ferric reducing ability of plasma, FRAP). We hypothesized heat acclimation training would result in a blunting of the acute oxidative stress response to exercise under hyperthermic conditions following the three-week acclimation period.

## Methods

### Participants

Participants’ characteristics are presented in Table 1. Untrained male (n = 25) and female (n = 13) participants between the ages of 19 and 40 years were recruited for the investigation. Inclusion criteria required successful screening using a Physical Activity Readiness Questionnaire (PAR-Q). Participants were required to be untrained due to the established relationship between exercise training and improved heat tolerance. Participants were excluded if they had engaged in a structured cardiovascular exercise program (3–5 days per week) within the three months prior to this study. Females without a regular menstrual cycle or those taking oral contraceptives within the previous eight months of the study were also excluded due to the potentially confounding effects of hormonal status. The investigation was approved by the Institutional Review Board (IRB) and the United States Army Medical Research and Development Command (USAMRMC) Human Research Protections Office (HRPO). Participants granted written consent prior to the study initiation.

**Table 1:** Participants’ characteristics.

Characteristic	PRE	POST
Males (n = 25)		
Age (yrs)	26 ± 7	
Body height (cm)	179.6 ± 8.4	
Body mass (kg)	85.2 ± 21.1	84.5 ± 21.0
W_max_ (W)	218 ± 46	251 ± 42
VO_2peak_ (mL·kg^-1^·min^-1^)	38.0 ± 7.2	41.5 ± 7.9
Females (n = 13)		
Age (yrs)	25 ± 4	
Body height (cm)	170.3 ± 1.6	
Body mass (kg)	62.9 ± 6.6	62.4 ± 5.3
W_max_ (W)	189 ± 86	199 ± 28
VO_2peak_ (mL·kg^-1^·min^-1^)	41.8 ± 6.5	41.7 ± 6.4

*N = 38; Values presented as mean ± SD.

### Measures

Fitness assessments were completed for all participants before and after the three-week training period. Fitness assessments consisted of body composition analysis and an aerobic capacity test (VO_2peak_). Body composition was quantified via hydrostatic weighing using an electronic load cell-based system (Exertech, Dresbach, MN). Residual lung volume estimates were determined, and final body density values were converted to percent body fat using the Siri equation (Siri, 1961). Peak oxygen uptake (VO_2peak_) was quantified during a graded exercise cycling protocol on an electronically braked cycle ergometer (Velotron, RacerMate, Seattle, WA). Starting at a workload of 95 W, participants increased exercise intensity by 35 W every three minutes until volitional fatigue was achieved. The highest oxygen uptake value obtained from 15 s intervals was used to determine VO_2peak_. Expired gases were quantified throughout the exercise test using a flow- and gas-calibrated metabolic cart (Parvomedics TrueOne 2400, Sandy, UT).

### Design and Procedures

**Figure 1:**
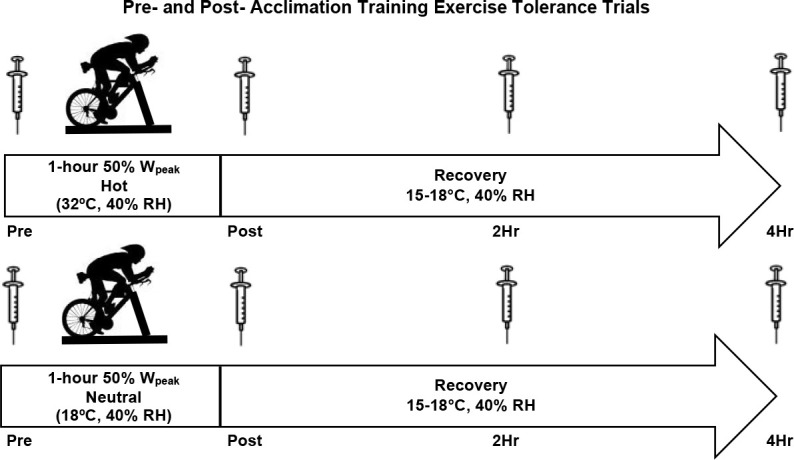
Experimental Design. Participants completed a pre- and post-acclimation trial separated by 15 exercise training sessions. Pre- and post-acclimation trials involved an identical 60-min bout of cycle ergometry at 50% of W_peak_. The experimental conditions included the following environmental conditions: Hot (33ºC, 40% Relative Humidity) and Room Temperature (20ºC, 40% Relative Humidity).

The investigation began with a baseline testing session to determine participants’ body composition and aerobic capacity (VO_2peak_). Participants were randomly assigned to a group in either a hot (33°C) or a room temperature (20°C) environment. On a separate visit, participants completed an exercise tolerance trial of cycling at 50% W_peak_ for duration of 60 min in the assigned environmental temperature. Participants continued the study with three weeks of acclimation training, which required cycling for 60 min at an intensity perceived as “hard”, over the course of 15 exercise sessions. After the completion of the acclimation training sessions, the exercise tolerance trial was repeated. Blood samples were collected prior to exercise (Pre), immediately post exercise (Post), two hours (2-HR), and four hours (4-HR) post exercise for the quantification of blood oxidative stress. The experimental design is presented in Figure 1.

Participants performed the exercise tolerance trials on the first and the last day of training. The exercise tolerance trials were conducted in a hot environment (33°C, 40% relative humidity) or a room temperature environment (20°C, 40% relative humidity), followed by a four-hour recovery period at 15–18°C. Prior to the trials, participants were asked to abstain from exercise for 48 hours, alcohol for 24 hours, and fast for a period of eight hours prior to testing. Before the first exercise tolerance trial, a 24-hour dietary recall was completed and replicated for the subsequent trial. Upon arrival, participants were fitted with skin temperature sensors on the chest and the back (SST-1 Skin Probe, Physitemp Instruments, Clifton NJ) and a rectal thermometer for the quantification of skin and core temperature (RET-1 Rectal Probe, Physitemp Instruments, Clifton, NJ). Heart rate monitoring (Polar V800, Polar Electronic, Lake Success, NY) was performed continuously throughout each trial. Participants completed 60 min of cycling at 50% W_peak_ on a cycle ergometer (Velotron) within their assigned environmental condition. Venous blood samples were collected at Pre, Post, 2-HR, and 4-HR after the exercise tolerance trials for the quantification of blood oxidative stress. During the exercise tolerance trials, whole blood samples were collected from the antecubital vein with sodium heparinized vacutainers. Venipuncture occurred Pre, Post, 2-HR, and 4-HR after the conclusion of the exercise tolerance trial. Blood samples were centrifuged at 1000 x g for 15 min at 4°C. Plasma aliquots were stored immediately at −80°C until the subsequent biochemical assay for oxidative damage and antioxidant biomarkers.

Participants arrived at the laboratory every weekday (Monday–Friday) for three weeks to complete the exercise training sessions. The training sessions consisted of 60 min of cycle ergometery at an intensity rated at 15 (hard) on the Borg Rating of Perceived Exertion (RPE) scale as previously described (Borg, 1973; McGlynn et al., 2022; Slivka et al., 2021). Participants were instructed to adjust exercise intensity to maintain an RPE of 15 throughout the training period. All training sessions were conducted in the assigned environmental temperature. The heart rate and core body temperature were continuously monitored during each training session and these data have been previously published (McGlynn et al., 2022; Slivka et al., 2021).

### Assays for Blood Oxidative Stress

A comprehensive panel of blood oxidative stress was used to quantify oxidative damage and antioxidant content. Lipid-based oxidative damage was measured through LOOH and 8-ISO, and protein-based oxidative damage was measured via PC and 3-NT. Plasma antioxidant capacity was measured by TEAC and FRAP. Blood plasma aliquots were subjected to a single freeze-thaw cycle and were kept on ice in a dark environment during the assay to prevent confounding results with environmental redox alterations.

A quantifiable colorimetric reaction was used for the quantification of TEAC. Plasma antioxidants scavenge exogenous radicals introduction of the reagent 2, 2’ azinobis 3-ethyl-benzothiazoline-6-sulfonic acid (ABTS). The formulation of the assay work solution began with 50 mM Glycine buffer and a 2.5 mM Trolox solution. Glycine-peroxidase concentrate was added to the 50 mM glycine buffer. The addition of ABTS solution and 22 mM H_2_O_2_ resulted in the final work solution. Final calculated TEAC values were derived from unknown samples using the water-soluble vitamin E analogue Trolox as a standard reference (Erel, 2004). A colorimetric reaction was used for the FRAP assay, which quantifies an exogenous ferric-to-ferrous tripyridyltriazine (TPTZ) reduction by antioxidants present within plasma. Within the FRAP assay, reduction of TPTZ is proportional to blood plasma antioxidant capacity and was measured by absorbance spectroscopy at 593 nm (Benzie and Strain, 1996).

Plasma LOOH was quantified using the ferrous oxidation-xylenol orange assay. Plasma samples were incubated in the presence or absence of a reducing agent and incubated with a colorimetric work solution containing ferrous ammonium sulfate, butylated hydroxytoluene, and xylenol orange. During the assay reaction, ferrous ions oxidize within the xylenol orange solution to form a quantifiable complex that was measured via absorbance spectroscopy at 595 nm. Final LOOH concentrations in unknown samples were derived using a cumene hydroperoxide standard reaction (Nourooz-Zadeh, 1999). Blood plasma 8-ISO concentrations were quantified by a commercially available specific immunoassay enzyme (EIA) (Cayman Chemical, Ann Arbor, MI) and assay procedures were performed according to manufacturer instructions (Montuschi et al., 1999).

Prior to the quantification of protein-based oxidative damage markers, sample protein concentrations were determined using the spectrophotometric Bradford method and then normalized for either PC or 3-NT assays based on the results of the Bradford’s study (Bradford, 1976). PC determination in blood plasma samples was performed by a commercially available ELISA kit according to manufacturer instructions (Biocell Corporation Ltd, Papatoetoe, NZ) (Buss et al., 1997). Samples were diluted based on anticipated ranges of 4–35 mg protein/ml. 3-NT content in blood plasma samples was determined via commercial ELISA and assayed according to manufacturer instructions (Cell Biolabs INC, San Diego, CA). Final concentrations for both PC and 3-NT assays were calculated based on spectrophotometric absorbance readings at 450 nm.

### Statistical Analysis

Blood oxidative stress responses were examined between the hot (33°C, 40% relative humidity) and room temperature (20°C, 40% relative humidity) groups using a 2 x 2 x 4 (temperature x trial x time) repeated-measures analysis of variance (ANOVA). Peak responses were determined by comparing the baseline values (Pre) with the highest elevated value of each blood oxidative stress marker (Peak). A 2 x 2 x 2 repeated-measures ANOVA (temperature x trial x time) was used to assess differences in peak responses between the temperature trials. Statistical procedures were performed using the Statistical Package for Social Sciences software (SPSS) Version 25.0 (Chicago, IL). All values are presented as means ± standard error mean (SEM). Significance was set *a priori* at *p* ≤ 0.05.

## Results

### Lipid Damage

**Figure 2:**
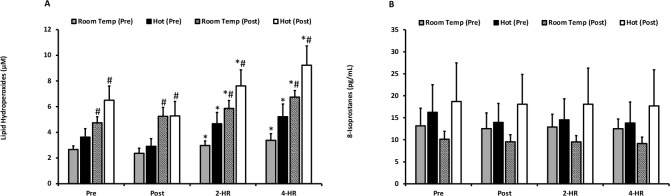
**(A) Plasma lipid hydroperoxides**. Lipid hydroperoxides are expressed as lipid hydroperoxide equivalents (µM) for exercise in a room temperature environment during the pre-acclimation trial (shaded bars), a hot environment during the pre-acclimation trial (black bars), a room temperature environment during the post-acclimation trial (dashed bars), and a hot environment during the post-acclimation trial (white bars). **(B) Plasma 8-Isoprostanes**. 8-Isoprostane values are expressed in standard comparison to 8-Isoprostanes protein content (pg/mL). * Significantly different from pre-exercise values. # Significantly different from the pre-acclimation trial. Means are expressed ± SEM.

Biomarkers for oxidative damage as quantified by LOOH and 8-ISO are presented in Figures 2A and 2B. For LOOH, no differences were observed between the hot and room temperature environments (*p* = 0.204). Exercise resulted in an elevation of LOOH at 2-HR (*p* = 0.001) and 4-HR (*p* ≤ 0.001). Pre and post acclimation trial-dependent differences (*p* ≤ 0.001) occurred, and a significant trial x time interaction effect was present (*p* = 0.018). Pairwise comparisons revealed that all LOOH values were higher in the second trial in both environmental temperatures (*p* ≤ 0.05), indicating that exercise training resulted in elevated blood plasma levels of LOOH. Peak LOOH responses were not different between environmental temperatures (*p* = 0.128), but a significant elevation occurred with exercise (*p* ≤ 0.001). Additionally, when examining peak responses an effect of the pre- and post-acclimation trial (*p* ≤ 0.001) occurred, as well as a trial x time interaction (*p* = 0.026). Further examination of the interaction effect for peak responses of LOOH also revealed that all post-acclimation training values were significantly higher than pre-acclimation training values (*p* ≤ 0.05). 8-ISO was unaffected by temperature (*p* = 0.287), acclimation training (*p* = 0.305), and exercise (*p* = 0.691). Peak responses revealed no differences between environmental temperature for LOOH (*p* = 0.128) or 8-ISO (*p* = 0.273).

### Protein Damage

**Figure 3:**
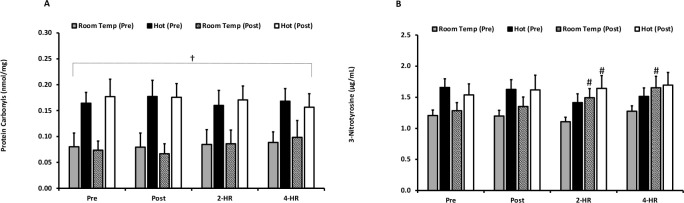
**(A) Protein Carbonyl** values are expressed in standard comparison to protein carbonyl equivalents (nmol/mg protein) for exercise in a room temperature environment during the pre-acclimation trial (shaded bars), a hot environment during the pre-acclimation trial (black bars), a room temperature environment during the post-acclimation trial (dashed bars), and a hot environment during the post-acclimation trial (white bars). (**B) Plasma Nitrotyrosine**. Nitrotyrosine values are expressed in standard comparison to nitrotyrosine protein content (ug/mL). # Significantly different from the pre-acclimation trial. † Significantly different between environmental temperatures. Means are expressed ± SEM.

Biomarkers for oxidative damage—as measured by PC and 3-NT—are presented in Figures 3A and 3B. The examination of PC revealed an inexplicable effect for temperature in PC values (*p* ≤ 0.001); however, no temperature x time interaction effect occurred (*p* = 0.195). PC was also unaffected by exercise (*p* = 0.351) and acclimation training (*p* = 0.393). When examining peak responses of PC, a main effect for temperature occurred (*p* = 0.001), but there was no temperature x time interaction effect (*p* = 0.437). Furthermore, there was no effect of acclimation training (*p* = 0.437). The peak response of PC was significantly elevated with exercise (*p* = 0.002). 3-NT was unaffected by environmental temperature (*p* = 0.253) and exercise (*p* = 0.053). A significant difference between pre- and post-acclimation trials (*p* = 0.026) was observed, as well as a trial x time interaction effect (*p* < 0.001). Pairwise comparisons revealed that 3NT was higher at 2-HR following exercise after three weeks of acclimation training under the hot environmental condition (*p* = 0.031). Additionally, 3NT was significantly higher at 2-HR (*p* = 0.001) and 4-HR (*p* = 0.009) after exercise under the room temperature condition following a three- week period of acclimation training. Examination of peak responses of 3-NT indicated that there were no differences between environmental temperatures (*p* = 0.204). However, an exercise-dependent increase was observed at peak (*p* < 0.001). Furthermore, an effect of trial (*p* = 0.048) and a trial x time interaction effect (*p* = 0.004) occurred. Pairwise comparisons determined that peak values were significantly higher in the room temperature trial following the three-week training period (*p* = 0.007).

### Antioxidant Capacity

**Figure 4:**
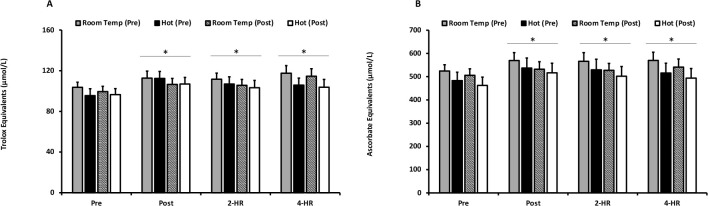
**(A) Trolox Equivalent Antioxidant Capacity**. Trolox Equivalent Antioxidant Capacity values are expressed as Trolox equivalent antioxidant capacity equivalents (umol/L) for exercise in a room temperature environment during the pre-acclimation trial (shaded bars), a hot environment during the pre-acclimation trial (black bars), a room temperature environment during the post-acclimation trial (dashed bars), and a hot environment during the post-acclimation trial (white bars). (**B) Ferric-reducing Ability of Plasma**. Ferric-reducing Ability of Plasma values are expressed as ascorbate equivalents (µmol/L). * Significantly different from pre-exercise values. Means are expressed ± SEM.

Mean responses for plasma antioxidant status assessed through the measurement of TEAC and FRAP are presented in Figures 4A and 4B. There was no effect of environmental temperature (*p* = 0.0542) or acclimation training (*p* = 0.587) on TEAC; however, an exercise effect was observed (*p* ≤ 0.001). TEAC was significantly elevated at Post (*p* ≤ 0.001), 2-HR (*p* ≤ 0.001), and 4-HR (*p* ≤ 0.001). Similarly, FRAP was unaffected by environmental temperature (*p* = 0.370) and acclimation training (*p* = 0.700), but increased significantly with exercise (*p* ≤ 0.001). FRAP increased at Post (*p* = 0.001) and remained elevated at 2-HR and 4-HR (*p* ≤ 0.001). Peak responses for TEAC were unaffected by temperature (*p* = 0.449). Additionally, peak responses for FRAP were not different between environmental temperatures (*p* = 0.30). The peak response for both TEAC and FRAP demonstrated a significant elevation in response to exercise (*p* ≤ 0.001).

## Discussion

Acute disruptions in redox balance due to exercise are beneficial for the development of exercise-induced adaptations (Gomez-Cabrera et al., 2008; Quindry et al., 2013). Importantly, previous investigations provide preliminary evidence that elevated core and/or environmental temperature can increase the blood oxidative stress response to exercise (Gomes et al., 2011; Laitano et al., 2010; Lee et al., 2019; McAnulty et al., 2005; Pilch et al., 2021; Quindry et al., 2013). The impact of an acclimation training period in a hot environment on the oxidative stress response to an acute exercise session, however, is unknown. Thus, the purpose of the current investigation was to examine the effect of an acclimation training period (15 exercise sessions) on the blood oxidative stress response to a single bout of exercise in a hot environment (33ºC). We hypothesized that an acclimation training period would blunt the elevated oxidative stress response typically observed following exercise performed in hot ambient temperatures. Contrary to our hypothesis, findings of the current investigation did not demonstrate a decrease in the blood oxidative stress response to a bout of exercise following the acclimation training period. Furthermore, oxidative stress was not elevated in response to exercise in a hot (33ºC), compared to a room temperature (20ºC) environment. Central to this methodological approach, we examined untrained individuals as acute exercise is well-demonstrated to result in the upregulation of cellular antioxidant fortifications, a fact that may otherwise be masked in athletic populations or those who regularly partake in exercise for improved health and fitness (Gomez-Cabrera et al., 2008).

LOOH and 8-ISO were selected as biomarkers of lipid damage for the current investigation. Importantly, both LOOH and 8-ISO perturbations have been observed in response to exercise, although few investigations have examined the impact of exercise in hot environments (Alessio et al., 2000; Ashton et al., 1999; Nieman et al., 2003; Quindry et al., 2003; Williamson-Reisdorph et al., 2021a). Among the few investigations to compare alterations in lipid peroxidation markers, Quindry et al. (2013) examined the effect of hot (32ºC) versus cold (7ºC) ambient temperatures. Findings demonstrated that moderate-intensity aerobic exercise (60% VO_2max_) elicited a significant elevation in LOOH one hour after exercising under the hot condition, although no differences were present when values were compared between the hot and neutral (20ºC) environmental conditions. Similarly, McAnulty et al. (2005) did not observe differences in the LOOH response between exercising in a hot (35ºC) or a thermo-neutral environment (25ºC). These findings are consistent with the hot-versus-neutral comparisons of the current investigation, in which no differences in LOOH were found between one hour of cycling at 50% W_peak_ under hot (33ºC) and room temperature (20ºC) environmental conditions. Although no differences were found between environmental temperatures following the acclimation training period, an effect of exercise training was observed with a greater elevation of LOOH following 15 exercise training sessions.

In contrast to LOOH, examination of the blood isoprostane responses to hyperthermic exercise shows that they are mixed (Laitano et al., 2010; McAnulty et al., 2005). Laitano et al. (2010) examined plasma isoprostanes corresponding to an elevated core body temperature of 1°C and 6°C from baseline and observed no significant elevations in either of the conditions. These prior findings are consistent with the current investigation, where no differences in exercise-induced responses under hot or room temperature conditions were found. However, McAnulty et al. (2005) observed a greater elevation of F_2_-isoprostanes (a similar biomarker to 8-ISO) following treadmill running at 50% VO_2max_, although it is possible that differences in the biochemical techniques to measure F_2_-isoprostanes and 8-ISO may account for the disparate outcomes. Importantly, the current investigation extends upon prior investigations by demonstrating that post exercise blood isoprostane levels were not impacted after the 15 session training period, a finding that contrasted another marker of lipid peroxidation, LOOH.

Protein damage, specifically free radical-mediated damage and modification of amino acid residues, was quantified through measurement of PC and 3-NT (Ahsan, 2013; Davies, 2016). Previous work has demonstrated an exercise-induced increase in circulating markers of free-radical mediated protein damage (Ballmann et al., 2014; Chirico et al., 2012; Wiecek et al., 2017; Woodside et al., 2014). The effect of hyperthermic exercise on blood protein carbonyl responses remains unclear and appears to be variably impacted by the mode of exercise in addition to the assay technique used to quantify biomarkers (Quindry et al., 2013; Souza-Silva et al., 2016; Sureda et al., 2015). Results of the current study demonstrated that post-exercise PC were not different following acute hyperthermic exercise. Given the importance of the methodological approach to measuring PC, it is notable that our findings are in agreement with those of Quindry et al. (2013) who utilized an identical assay technique. In that prior study, PC were not impacted by one hour of exercise (60% VO_2max_) at hot ambient temperature (32ºC) (Quindry et al., 2013). Furthermore, Sureda et al. (2015) observed no differences in PC responses to 45 min of treadmill exercise at 75–80% VO_2max_, indicating that PC responses may not be sensitive to hyperthermic conditions when examined in these types of exercise challenges (Sureda et al., 2015). Central to the current investigation, we are the first to demonstrate that blood PC responses are also not impacted by an acute bout of exercise either before or after a 15 session training intervention.

The current investigation also examined protein damage through the quantification of 3-NT, a relatively novel biomarker as applied to exercise and oxidative stress investigations. Similarly to our findings of blood PC, 3-NT was not altered following exercise in either the hot or neutral ambient temperature conditions. Moreover, a novel facet of the current investigation was the examination of post exercise values of 3-NT before and after a 15 session training intervention. By comparison, in a previous work which examined the impact of four weeks of interval training under hyperthermic conditions, protein oxidation was blunted (Sureda et al., 2015), although it seems possible that the variable intensity of the prior exercise protocol may have accounted for diffent outcomes as compared to the current study. Indeed, due to the lack of temperature effects is this investigation, it is unclear whether an acclimation training period could blunt oxidative stress responses with respect to this marker for protein oxidative damage. Further research is necessary to clarify the impact of hyperthermic exercise training on protein oxidation responses to traditional exercise training (i.e., exercise prescriptions for healthy adults) and to extended duration events (i.e., ultramarathons, etc.).

Blood plasma levels of TEAC and FRAP, index measures of antioxidant quenching capacity in diverse biological fluids, are known to elevate during fatiguing exercise performed under normothermic conditions. Alteration in blood antioxidant quenching capacity, while sometimes viewed as counter intuitive, occurs in response to the acceleration of purine metabolism following fatiguing exercise. That is, exercise results in the enzymatic production of the free radical superoxide and subsequently produces uric acid, the most plentiful water-soluble antioxidant in blood plasma (Ballmann et al., 2014; Peters et al., 2018; Quindry et al., 2013; Williamson-Reisdorph et al., 2021a, 2021b). Consistent with this biochemical understanding, findings of the current investigation demonstrated an exercise-induced elevation in both TEAC and FRAP independent of the ambient temperature.

Regarding the role of hyperthermia, there are limited investigations examining TEAC and FRAP responses to exercise. Quindry et al. (2013) examined exercise-induced responses to moderate-intensity aerobic exercise and determined that TEAC and FRAP were elevated to a greater extent when exercise was performed under hyperthermic conditions, indicating greater antioxidant quenching capacity due to the metabolic production of uric acid (Quindry et al., 2013). In contrast to the current findings, a prior investigation which examined responses of reduced glutathione (GSH), oxidized glutathione (GSSG), and superoxide dismutase (SOD) to moderate-intensity exercise, observed an elevation in antioxidant capacity (Laitano et al., 2010); similarly, Sureda et al. (2015) as quantified by the post exercise activity of the enzymatic antioxidant catalase. They observed elevations in antioxidant status following hyperthermic exercise, further supporting prior work which suggests that exercise-induced oxidative stress in hot temperatures results in a greater elevation of antioxidant capacity within the blood. However, differences in the exercise modality (interval training in the prior study versus moderate continuous training in the current investigation) could account for contradictory outcomes between research groups. Indeed, in the current investigation we did not observe a hyperthermic-induced elevation in oxidative stress, although, elevated metrics of antioxidant capacity were observed following both normothermic and hyperthermic environmental challenges. Given the lack of changes in TEAC and FRAP at the time points before and after the 15 session training period, it is difficult to ascertain the potential impact on these blood measures. Nonetheless, it seems plausible that combining the current approach with prior experimental designs which utilized variable intensity exercise training may be revealing. Accordingly, we recommend the inclusion of calorically similar training protocols using moderate continuous versus high intensity interval approaches to further examine the potential role of acclimation training in these important markers of blood antioxidant status.

The current investigation employed a novel approach to examine a holistic panel of oxidative stress, which included two biomarkers of lipid damage, protein damage, and antioxidant capacity. Importantly, based on the current findings, an exercise training period at hyperthermic temperatures does not blunt the blood oxidative stress responses to an acute bout of exercise. Furthermore, the results of the current study add to the growing body of literature regarding blood oxidative stress responses to acute exercise under hyperthermic conditions. Biomarkers of lipid damage, protein oxidation, and/or antioxidant capacity were not altered by acute exercise under hyperthermic conditions within the current investigation. Limitations of the current investigation include the absence of the analysis of sex-dependent differences and physical activity monitoring during the three-week training period. The sample size in the current investigation did not allow the analyses of sex-dependent differences due to a lack of power. Future research should emphasize the effects of various exercise training interventions (e.g., intensities and modalities) to better understand the impact of acclimation training on the blood oxidative stress response. Furthermore, future investigations should determine whether there are sex- dependent differences in the blood oxidative stress response to acute hyperthermic exercise, due to the established relationship between estrogen concentrations and altered thermoregulatory responses, and whether heat acclimation training offers advantages to oxidative stress responses in more extreme forms of exercise. As a final consideration, given the link between oxidative stress as an adaptive stimulus to exercise training (Gomez-Cabrera et al., 2008), the current pre-to-post changes in peak aerobic capacity were non-significantly altered in both females and males. While negative outcomes for both oxidative stress and peak aerobic capacity are inexplicable within the current study design, directed examination of these relationships should be central to follow-up investigations. Finally, we also recommend the future investigations perform a comparison between moderate intensity and interval training approaches that are matched for total energy expenditure as a way of better understanding the role of the exercise stimulus on training adaptations and post exercise oxidative stress responses.

## Author Contributions

Conceptualization: D.S., B.C.R. and J.C.Q.; methodology: T.S.Q., K.S.C., S.C.G., K.G.T., J.C.,W.H., D.S., B.C.R. and J.C.Q.; validation: T.S.Q. and J.C.Q.; formal analysis: C.M.W.-R., D.S. and J.C.Q.; investigation: D.S., B.C.R. and J.C.Q.; resources: D.S., T.S.Q., J.C., W.H., B.C.R. and J.C.Q.; data curation: T.S.Q., W.H., D.S. and J.C.Q.; writing—original draft preparation: J.C.Q. and C.M.W.-R.; writing—review & editing: C.M.W.-R., J.C.Q., D.S. and B.C.R.; visualization: D.S., B.C.R. and J.C.Q.; supervision: D.S., B.C.R. and J.C.Q.; project administration: T.S.Q., W.H., D.S., B.C.R. and J.C.Q.; funding acquisition: D.S., B.C.R. and J.C.Q. All authors have read and agreed to the published version of the manuscript.

## ORCID iD

John Quindry: 0000-0003-4655-0086
